# Treatment and Outcomes for Patients With Inadequate Lymphadenectomy After Resection of Stage II Small Bowel Adenocarcinoma

**DOI:** 10.1002/jso.70151

**Published:** 2025-12-09

**Authors:** Jackson A. Baril, Karl Y. Bilimoria, Eugene P. Ceppa, Michael G. House, Thomas K. Maatman, Alexandra M. Roch, Anthony D. Yang, C. Max Schmidt, Ryan J. Ellis

**Affiliations:** ^1^ Surgical Outcomes and Quality Improvement Center (SOQIC), Department of Surgery Indiana University School of Medicine Indianapolis Indiana USA; ^2^ Department of Surgery, Division of Surgical Oncology Indiana University School of Medicine Indianapolis Indiana USA

**Keywords:** adjuvant chemotherapy, Intestinal Neoplasms, lymphadenectomy, Retrospective Study, small Intestine/surgery

## Abstract

**Background and Objectives:**

Adjuvant chemotherapy (AC) is considered for patients with stage II small bowel adenocarcinoma (SBA) with an inadequate lymphadenectomy; however, the prognostic role of additional high‐risk features (T4 primary, positive resection margin, poorly differentiated tumor, or lymphovascular invasion) is unknown. The objectives were to describe utilization of AC among patients with stage II SBA with inadequate lymphadenectomy, identify factors associated with receipt of AC, and examine the association between AC and survival stratified by presence of additional high‐risk features.

**Methods:**

Patients with stage II SBA were identified using the National Cancer Database from 2004 to 2021. Inadequate lymphadenectomy was defined < 5 lymph nodes duodenal tumors and < 8 lymph nodes other sites.

**Results:**

Of 1765 patients with stage II SBA and an inadequate lymphadenectomy, 525 (29.8%) received AC. T4 primary, poor grade tumor, and positive resection margin were associated with receiving AC. Receipt of AC was associated with improved 5‐year survival in patients with additional high‐risk features (49.9% vs 31.4%; HR 0.62, 95%CI 0.48–0.79) but not in patients without additional high‐risk features (67.1% vs. 53.2%; HR 0.83, 95%CI 0.55–1.24).

**Conclusions:**

Receipt of AC was associated with improved survival in patients with inadequate lymphadenectomy and any additional high‐risk feature. Multiple variables may be considered in decisions regarding AC.

## 1 Introduction

Small bowel adenocarcinoma (SBA) is rare and accounts for around 1 in 3 small bowel malignancies. SBA has a poor prognosis with a median overall survival (OS) of 12.8 months for duodenal tumors and 40.4 months for jejunal tumors [1, 2, 3]. In 2024, an estimated 12,440 patients will be diagnosed with SBA in the United States and over 2000 will die from the disease [4]. Patients with Crohn Disease, ulcerative colitis, Lynch syndrome, familial adenomatous polyposis, and Celiac disease are at increased risk of developing SBA [5, 6, 7, 8, 9, 10]. SBA rarely is detected on cross‐sectional imaging and may be found incidentally during resections for stricture, bowel obstruction, or fistulizing disease [9, 11]. Without a suspected diagnosis of malignancy, an oncologic resection might not be performed leading to inadequate lymphadenectomy. Due to the rarity of SBA, limited prospective data are available to guide treatment in patients with an early stage primary and inadequate lymphadenectomy and thus some recommendations are extrapolated from colorectal adenocarcinoma data [12]. However, current guidelines for treatment of SBA recommend adjuvant chemotherapy for stage III disease and to consider adjuvant chemotherapy in stage II patients with other high‐risk features such as T4 primary tumor, positive resection margin, poor differentiation, tumor perforation, lymphovascular or perineural invasion, and inadequate lymphadenectomy [13, 14].

According to current guidelines, an inadequate lymphadenectomy is defined as removal of fewer than 5 lymph nodes for duodenal tumors and fewer than 8 lymph nodes for tumors of the ileum or jejunum [14]. Node‐positive disease is known to have worse outcomes, thus adequate staging for treatment sequencing is critical [3, 15, 16]. Studies have suggested varying cutoffs for adequate lymphadenectomy to optimize staging and survival: removal of 5 to 8 lymph nodes for tumors of the duodenum and 8 to 16 lymph nodes for tumors of the jejunum or ileum [17, 18, 19, 20]. It is hypothesized that an adequate lymphadenectomy misses nodal disease leading to risk of understaging [18]. Thus, patients with inadequate lymphadenectomy who forego adjuvant chemotherapy are at risk for undertreatment of occult nodal disease.

Previous studies have examined the optimal number of lymph nodes to adequately stage patients with small bowel adenocarcinoma; however, whether an inadequate lymphadenectomy necessitates adjuvant chemotherapy in the presence or absence of additional high‐risk features has not been well described. The objectives of this study are to (1) describe the rate of adjuvant chemotherapy use among patients with stage II small bowel adenocarcinoma, (2) identify factors associated with receipt of adjuvant chemotherapy and (3) examine the association between chemotherapy and overall survival among patients with inadequate lymphadenectomy stratified by the presence or absence of additional high‐risk features.

## 2 Materials and Methods

### 2.1 Data Source

This retrospective cohort study of the National Cancer Database (NCDB) 2021 participant user file (PUF) examined patients diagnosed with small bowel adenocarcinoma from January 1st, 2004, to December 31st, 2021. The NCDB is a registry of patients treated at Commission on Cancer (CoC) affiliated sites across the United States and includes data from over 70% of invasive cancer diagnoses nationwide [21]. The project is not human subjects research and thus exempt from institutional review board (IRB) review.

### 2.2 Study Population

Patients aged 18 and older with confirmed pathologic diagnosis of stage II small bowel adenocarcinoma disease who underwent upfront, curative‐intent surgery and were found to have an inadequate lymphadenectomy were included. An inadequate lymphadenectomy was defined as fewer than 5 lymph nodes retrieved for tumors of the duodenum and fewer than 8 lymph nodes for tumors of the jejunum or ileum. Small bowel adenocarcinomas were identified using International Classification of Diseases for Oncology (ICD‐O‐3) histology codes for non‐mucinous adenocarcinomas 8140, 8143–8145, 8210, 8211, 8255, 8261–8263, and 8490 in locations C17.0, 17.1, 17.2, and 17.9. Patients with missing pathologic staging data, pathologic stage other than stage II, neoadjuvant therapy, missing number of nodes examined, or missing post‐operative treatment data were excluded. In assessing factors associated with receipt of chemotherapy, patients with documented refusal of chemotherapy were excluded. In assessing survival, patients diagnosed in year 2021 and patients who died or were censored within 30 days of surgery were excluded.

### 2.3 Outcomes and Covariates

Outcomes of interest were receipt of adjuvant chemotherapy within 180 days after surgery and overall survival among patients undergoing surgery for stage II small bowel adenocarcinoma. Overall survival was defined as time from diagnosis to last follow up or death. High‐risk features were defined as T4 primary tumor, positive resection margin, poor differentiation and lymphovascular invasion. Other key covariates contained within the NCDB included basic social and demographic variables, Charlson‐Deyo Comorbidity Index, and hospital characteristics. Average annual hospital volume was defined as the number of patients treated for SBA divided by the number of years the hospital participated in the NCDB. Staging was defined using the American Joint Committee on Cancer (AJCC) 8th edition TNM staging.

### 2.4 Statistical Analysis

Factors associated with receipt of chemotherapy after upfront resection were assessed using multivariable logistic regression. Models were constructed with all sociodemographic variables and clinically relevant variables included. Missing data were classified as a separate category for multivariable logistic regression analysis. Postoperative survival was assessed using log‐rank testing. Multivariable Cox proportional hazard models were used to assess receipt of adjuvant chemotherapy and postoperative survival in patients with inadequate lymphadenectomy, stratified by the presence or absence of additional high‐risk features. To adjust for immortal time bias, a Cox proportional hazard model with landmark analysis was employed, excluding patients who died or were censored within 180 days. A significance level of α < 0.05 was used for statistical tests. Analyses were completed using Stata® Standard Edition version 18.5 (StataCorp, College Station, TX).

## 3 Results

### 3.1 Study Cohort

**Figure 1:**
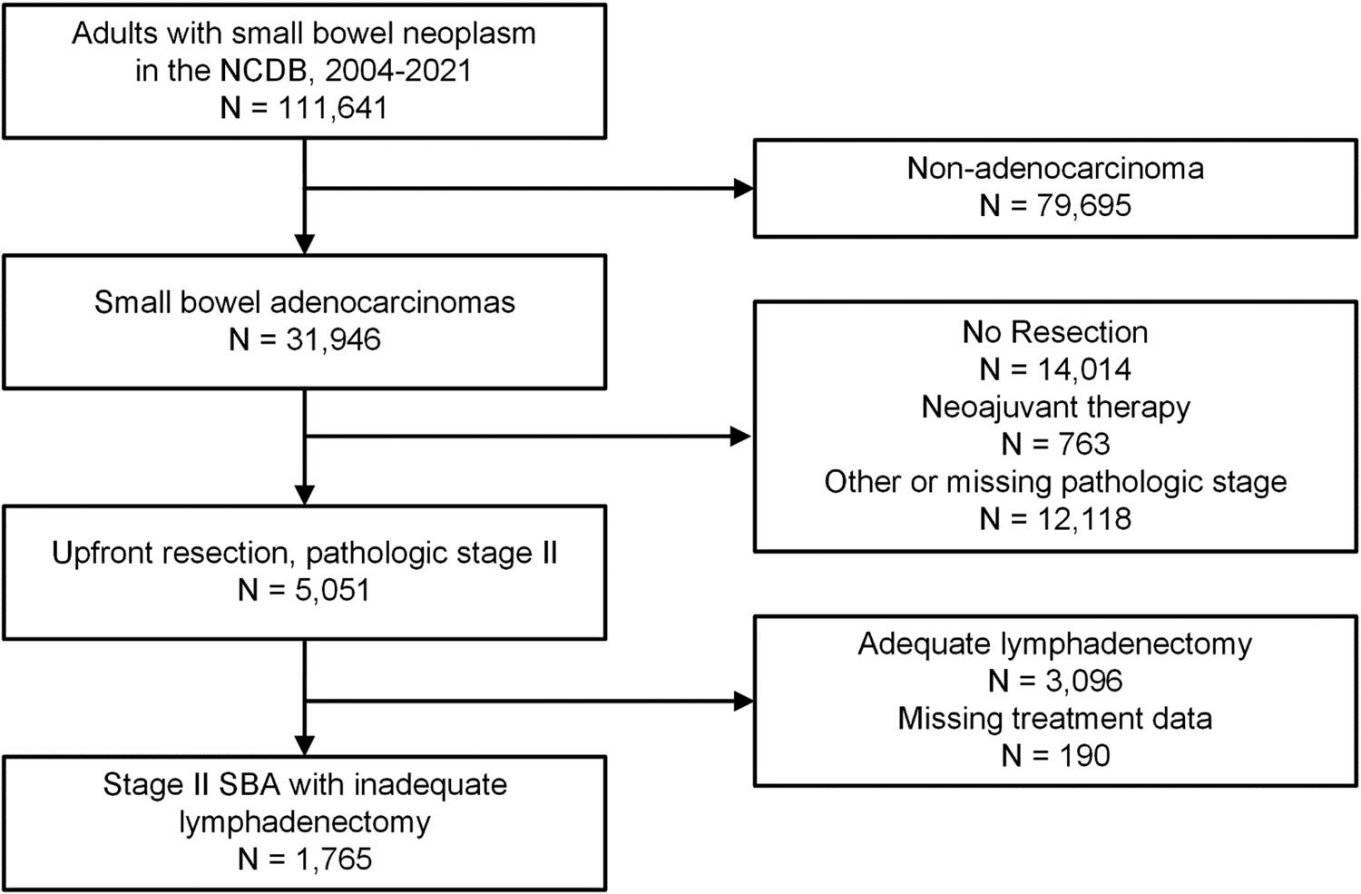
Patient cohort flow diagram. NCDB = National Cancer Database.

Of 5051 patients who underwent resection for stage II SBA, 1765 (34.9%) patients had an inadequate lymphadenectomy (Figure 1). An inadequate lymphadenectomy occurred in 350 (20.9%) of duodenal tumors, 569 (49.1%) of jejunal tumors, 274 (32.7%) of ileal tumors, and 572 (61.1%) of other or non‐specified sites in the small intestine. Among all patients with stage II SBA and an inadequate lymphadenectomy, the median age was 68 years, 815 (46.2%) were female, and 1013 (57.4%) had at least one additional high‐risk feature. Most of the cohort was non‐Hispanic white (*n* = 1224, 69.4%), had a Charlson‐Deyo Score of 0 (*n* = 1218, 69.0%), and had Medicare insurance (*n* = 994, 56.3%). Most tumors were T3 (*n* = 1057, 59.9%) and moderate grade (*n* = 1106, 62.7%). Additional cohort characteristics are described in Table 1.

**Table 1:** Patient, tumor, and treating facility characteristics of patients with pathologic stage II small bowel adenocarcinoma with an inadequate lymphadenectomy.

*N* = 1765	Number (%)
*Patient and Tumor Characteristics*	
Sex	
Female	815 (46.2)
Male	950 (53.8)
Age (yrs)	
< 50	201 (11.4)
50–59	295 (16.7)
60–69	456 (25.8)
70–79	473 (26.8)
80+	340 (19.3)
Race and Ethnicity	
Non‐Hispanic White	1224 (69.4)
Non‐Hispanic Black	320 (18.1)
Hispanic	85 (4.8)
Asian	38 (2.2)
Other or Unknown	98 (5.6)
Median Household Income	
< $46,277	272 (15.4)
$46,277–$57,856	362 (20.5)
$57,857–$74,062	374 (21.2)
$74,063+	545 (30.9)
Unknown	212 (12.0)
% Local No High School Diploma	
< 5%	330 (18.7)
5.0–9.0%	475 (26.9)
9.1–15.2%	438 (24.8)
15.3% and up	316 (17.9)
Unknown	206 (11.7)
Insurance Status	
Private Insurance	570 (32.3)
Medicare	994 (56.3)
Uninsured/Medicaid	148 (8.4)
Other or Unknown	53 (3.0)
Charlson–Deyo Score	
0	1218 (69.0)
1	357 (20.2)
2+	190 (10.8)
Year of Diagnosis	
2004–2008	466 (26.4)
2009–2013	513 (29.1)
2014–2018	502 (28.4)
≥ 2018	284 (16.1)
Tumor Location	
Duodenum	350 (19.8)
Jejunum	569 (32.2)
Ileum	274 (15.5)
Other, Small Intestine	572 (32.4)
T Stage	
3	1057 (59.9)
4	630 (35.7)
Unknown	78 (4.4)
Lymphovascular Invasion	
Absent	783 (44.4)
Present	293 (16.6)
Unknown	689 (39.0)
Histologic Grade	
Well	194 (11.0)
Moderate	1106 (62.7)
Poor	400 (22.7)
Unknown	65 (3.7)
Resection Margin	
Negative	1524 (86.4)
Positive	205 (11.6)
Unknown or Indeterminate	36 (2.0)
*Hospital Characteristics*	
Hospital Type	
Academic	535 (30.3)
Non‐Academic	1230 (69.7)
Hospital Annual Volume Quartile	
Q1 (< 4)	539 (30.5)
Q2 (4–8)	506 (28.7)
Q3 (9–15)	411 (23.3)
Q4 (≥ 16)	309 (17.5)
Hospital Region	
New England	80 (4.5)
Middle Atlantic	284 (16.1)
South Atlantic	429 (24.3)
East North Central	273 (15.5)
East South Central	119 (6.7)
West North Central	127 (7.2)
West South Central	136 (7.7)
Mountain	71 (4.0)
Pacific	184 (10.4)
Unknown	62 (3.5)

### 3.2 Factors Associated With Receipt of AC

Of patients with an inadequate lymphadenectomy, 525 (29.8%) received AC overall including 364 (35.9%) with any high‐risk feature and 161 (21.4%) with no high‐risk features. Most patients, 351 (66.9%), received multiagent AC, while 145 (27.6%) received single agent AC and 29 (5.5%) had unknown regimens. Several clinicopathologic features were associated with receipt of AC including T4 primary tumor (vs. T3 primary tumor, aOR 1.97; 95%CI 1.50–2.57; *p* < 0.001), poor grade tumor (vs. moderate, aOR 1.42, 95%CI 1.07–1.89; *p* = 0.015), and positive resection margin (vs. negative margin, aOR 1.62, 95%CI 1.12–2.35; *p* = 0.011). Older patients were less likely to receive chemotherapy including those aged 70–79 (vs. age 50–59, aOR 0.32, 95%CI 0.20–0.50; *p* < 0.001) and 80+ (vs. age 50–59, aOR 0.07, 95%CI 0.04–0.14, *p* < 0.001). Patients living in areas where a higher percentage (≥ 15.3%) of adults with no high‐school diploma were less likely to receive chemotherapy (vs. < 5%, aOR 0.50, 95%CI 0.30–0.83; *p* = 0.007). A numerically higher rate of AC use was found in later years of diagnosis and for jejunal tumors (vs. duodenal), but did not reach statistical significance. Hospital type (academic or non‐academic) and average annual volume of small bowel adenocarcinomas were not associated with receipt of AC (Table 2).

**Table 2:** Factors associated with receipt of adjuvant chemotherapy among patients with stage II small bowel adenocarcinoma with an inadequate lymphadenectomy.

	Receipt of adjuvant chemotherapy (%)	aOR (95% confidence interval)	*p* value
Patient and Tumor Characteristics			
Sex			
Female	28.8	1	Ref
Male	30.5	1.01 (0.79–1.28)	0.961
Age (yrs)			
< 50	51.7	1.11 (0.71–1.76)	0.644
50–59	44.4	1	Ref
60–69	37.3	0.73 (0.50–1.06)	0.100
70–79	21.4	0.32 (0.20–0.50)	< 0.001
80+	5.6	0.07 (0.04–0.14)	< 0.001
Race and Ethnicity			
Non‐Hispanic White	29.2	1	Ref
Non‐Hispanic Black	33.4	1.05 (0.73–1.50)	0.800
Hispanic	29.4	0.71 (0.38–1.32)	0.273
Asian	29.0	0.97 (0.39–2.43)	0.956
Other or Unknown	25.5	0.93 (0.52–1.68)	0.822
Median Household Income			
< $46,277	29.4	1	Ref
$46,277–$57,856	26.5	0.93 (0.61–1.41)	0.724
$57,857–$74,062	30.5	0.86 (0.55–1.32)	0.487
$74,063+	32.1	0.73 (0.45–1.19)	0.210
% Local No High School Diploma			
< 5%	35.5	1	Ref
5.0–9.0%	31.1	0.83 (0.58–1.20)	0.323
9.1–15.2%	27.4	0.65 (0.42–1.01)	0.056
15.3% and up	25.9	0.50 (0.30–0.83)	0.007
Insurance Status			
Private Insurance	43.3	1	Ref
Medicare	21.4	0.95 (0.68–1.34)	0.775
Uninsured/Medicaid	37.2	0.78 (0.52–1.17)	0.227
Other/Unknown	18.9	0.50 (0.21–1.15)	0.103
Charlson–Deyo Score			
0	32.4	1	Ref
1	24.7	0.82 (0.60–1.14)	0.235
2+	22.6	0.70 (0.45–1.08)	0.106
Year of Diagnosis			
2004–2008	24.9	0.79 (0.47–1.34)	0.386
2009–2013	27.7	0.69 (0.47–1.00)	0.051
2014–2018	34.1	0.92 (0.63–1.34)	0.659
≥ 2018	33.8	1	Ref
Tumor Location			
Duodenum	24.6	1	Ref
Jejunum	36.6	1.37 (0.96–1.93)	0.080
Ileum	26.3	1.06 (0.71–1.60)	0.765
Other, Small Intestine	27.8	0.94 (0.66–1.33)	0.725
T Stage			
3	25.0	1	Ref
4	38.3	1.96 (1.50–2.57)	< 0.001
Lymphovascular Invasion			
Absent	30.4	1	Ref
Present	38.9	1.15 (0.82–1.62)	0.410
Histologic Grade			
Well	24.2	0.76 (0.50–1.15)	0.191
Moderate	29.0	1	Ref
Poor	35.5	1.42 (1.07–1.89)	0.015
Resection Margin			
Negative	28.9	1	Ref
Positive	37.1	1.62 (1.12–2.35)	0.011
Unknown or Indeterminate	25.0	1.53 (0.62–3.78)	0.349
Hospital Characteristics			
Hospital Type			
Academic	32.0	1	Ref
Non‐Academic	28.8	1.00 (0.71–1.41)	0.989
Hospital Annual Volume Quartile			
Q1 (< 4)	30.6	0.92 (0.59–1.43)	0.721
Q2 (4–8)	26.3	0.77 (0.51–1.16)	0.212
Q3 (9–15)	30.7	0.94 (0.66–1.33)	0.747
Q4 (≥ 16)	33.7	1	Ref
Hospital Region			
New England	37.5	1	Ref
Middle Atlantic	28.9	0.66 (0.36–1.23)	0.195
South Atlantic	30.1	0.65 (0.37–1.16)	0.144
East North Central	27.1	0.63 (0.34–1.14)	0.127
East South Central	23.5	0.45 (0.21–0.93)	0.031
West North Central	30.7	0.77 (0.40–1.48)	0.428
West South Central	26.5	0.56 (0.29–1.08)	0.082
Mountain	35.2	0.96 (0.50–1.87)	0.912
Pacific	24.5	0.62 (0.33–1.15)	0.129

Abbreviation: aOR = adjusted odds ratio.

### 3.3 Adjuvant Chemotherapy and Postoperative Survival

**Figure 2:**
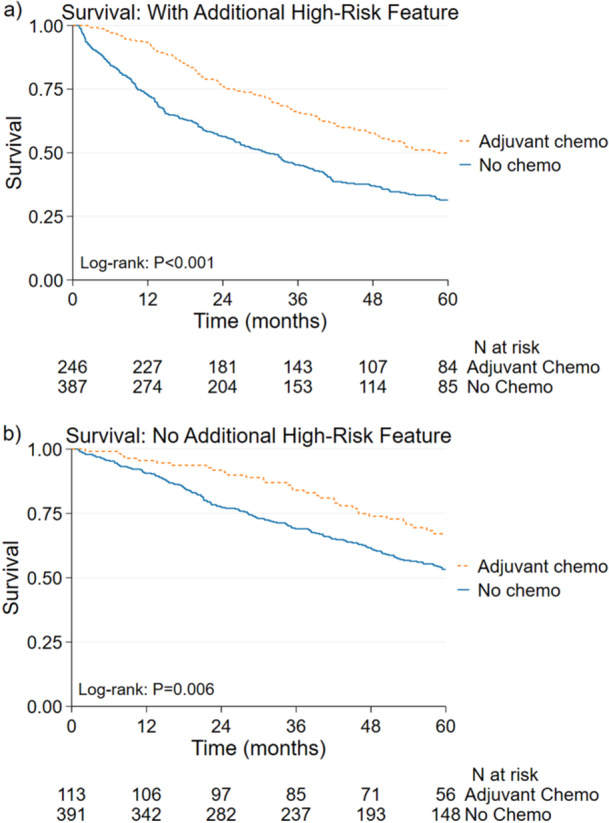
Kaplan‐Meier survival curves for patients with stage II small bowel adenocarcinoma and inadequate lymphadenectomy (a) with at least one additional high‐risk feature and (b) with no additional high‐risk feature. High‐risk features include T4 primary tumor, positive resection margin, poor differentiation, and lymphovascular invasion.

Receipt of AC, compared with no receipt of AC, was associated with improved 5‐year postoperative survival on unadjusted log‐rank test (*p* < 0.001) and adjusted Cox model (55.3% vs. 42.3%; Hazard Ratio for mortality (HR) 0.67, 95%CI 0.55–0.83; *p* < 0.001). When stratified by the presence of additional high‐risk features, receipt of AC, compared with no receipt of AC, was associated with improved 5‐year survival for patients with any additional high‐risk feature on adjusted Cox model (49.9% vs. 31.4%; HR 0.62, 95%CI 0.48–0.79; *p* < 0.001 Figure 2a). Among patients without additional high‐risk features, AC was associated with improved 5‐year survival on unadjusted log‐rank (*p* = 0.006) but not on adjusted Cox model (67.1% vs. 53.2%; HR 0.83, 95%CI 0.55–1.24; *p* = 0.363 Figure 2b, Table 3). Two sensitivity analyses (1) excluding patients 80 years and older and (2) excluding patients diagnosed before 2011 were performed with similar results on the association of adjuvant chemotherapy with survival. A separate landmark analysis excluding patients who died or were censored within 180 days of surgery was performed to adjust for immortal time bias and the Cox model had similar results (Supplemental Table 1).

**Table 3:** Cox proportional hazard ratios for mortality among patients with pathologic stage II small bowel adenocarcinoma and inadequate lymphadenectomy with and without at least one additional high‐risk feature.

	With additional high‐risk feature(s) *N* = 633 h (95% CI)	*p* value	Without additional high‐risk feature *N* = 504 h (95% CI)	*p* value
*Patient and Tumor Characteristics*				
Post‐operative Chemotherapy				
No	1	Ref	1	Ref
Yes	0.62 (0.48–0.79)	< 0.001	0.83 (0.55–1.24)	0.363
Sex				
Female	0.76 (0.60–0.95)	0.016	1.38 (1.02–1.86)	0.038
Male	1	Ref	1	Ref
Age (yrs)				
< 50	0.90 (0.54–1.49)	0.683	0.60 (0.29–1.25)	0.174
50–59	1	Ref	1	Ref
60–69	1.18 (0.82–1.71)	0.372	0.94 (0.56–1.57)	0.805
70–79	1.09 (0.71–1.67)	0.703	1.19 (0.67–2.13)	0.549
80+	1.60 (1.01–2.53)	0.046	2.33 (1.31–4.18)	0.004
Race and Ethnicity				
Non‐Hispanic White	1	Ref	1	Ref
Non‐Hispanic Black	1.25 (0.90–1.74)	0.190	1.03 (0.69–1.55)	0.878
Hispanic	1.40 (0.80–2.45)	0.233	1.19 (0.53–2.69)	0.669
Asian	0.82 (0.28–2.40)	0.721	1.30 (0.30–5.67)	0.724
Other or Unknown	1.72 (1.14–2.61)	0.010	1.34 (0.70–2.58)	0.375
Median Household Income				
< $46,277	1	Ref	1	Ref
$46,277–$57,856	0.82 (0.57–1.28)	0.441	1.11 (0.66‐‐1.89)	0.684
$57,857–$74,062	0.82 (0.54–1.23)	0.330	1.05 (0.61–1.81)	0.870
$74,063+	0.74 (0.48–1.14)	0.171	1.31 (0.72‐2.38)	0.369
% Local No High School Diploma				
< 5%	1	Ref	1	Ref
5.0–9.0%	0.77 (0.55–1.08)	0.133	0.90 (0.55–1.45)	0.658
9.1–15.2%	0.59 (0.39–0.88)	0.010	1.02 (0.59–1.75)	0.951
15.3% and up	0.0.69 (0.44–1.09)	0.114	0.82 (0.43–1.56)	0.544
Insurance Status				
Private Insurance	1	Ref	1	Ref
Medicare	1.05 (0.75–1.48)	0.769	1.24 (0.80–1.91)	0.339
Uninsured/Medicaid	1.38 (0.87–2.17)	0.167	1.89 (1.03–3.46)	0.039
Other/Unknown	1.09 (0.54–2.19)	0.805	2.70 (1.12–6.47)	0.026
Charlson–Deyo Score				
0	1	Ref	1	Ref
1	1.45 (1.11–1.90)	0.007	1.07 (0.74–1.55)	0.719
2+	2.05 (1.42–2.96)	< 0.001	1.43 (0.85–2.43)	0.181
Year of Diagnosis				
2004–2008	1.33 (0.76–2.32)	0.324	1.12 (0.58–2.17)	0.733
2009–2013	1.10 (0.71–1.70)	0.683	0.98 (0.51–1.88)	0.941
2014–2018	1.08 (0.70–1.67)	0.718	0.91 (0.47–1.80)	0.795
≥ 2018	1	Ref	1	Ref
Tumor Location				
Duodenum	1	Ref	1	Ref
Jejunum	0.69 (0.46–1.03)	0.071	0.58 (0.36–0.93)	0.023
Ileum	0.97 (0.63–1.50)	0.899	0.67 (0.39–1.15)	0.149
Other, Small Intestine	1.13 (0.77–1.66)	0.519	0.97 (0.62–1.51)	0.889
*Hospital Characteristics*				
Hospital Type				
Academic	1	Ref	1	Ref
Non‐Academic	1.16 (0.84–1.60)	0.379	1.48 (0.97–2.26)	0.067
Hospital Annual Volume Quartile				
Q1 ( < 4)	1.02 (0.73–1.42)	0.899	1.20 (0.65–2.19)	0.564
Q2 (4–8)	0.94 (0.64–1.38)	0.765	1.42 (0.79–2.52)	0.238
Q3 (9–15)	0.90 (0.60–1.35)	0.599	1.01 (0.56–1.82)	0.964
Q4 ( ≥ 16)	1	Ref	1	Ref
Hospital Region				
New England	1	Ref	1	Ref
Middle Atlantic	1.11 (0.61–2.03)	0.734	1.96 (0.74–5.18)	0.175
South Atlantic	1.12 (0.61–2.06)	0.704	1.42 (0.55–3.73)	0.466
East North Central	0.80 (0.43–1.48)	0.484	2.12 (0.81–5.58)	0.127
East South Central	0.87 (0.44‐1.74)	0.695	1.24 (0.42–3.68)	0.694
West North Central	1.06 (0.56–2.03)	0.848	2.25 (0.80–6.33)	0.126
West South Central	0.92 (0.48–1.78)	0.814	1.56 (0.55‐4.45)	0.402
Mountain	1.19 (0.54–2.64)	0.661	1.40 (0.40–4.88)	0.594
Pacific	0.74 (0.38–1.43)	0.365	1.42 (0.51–3.98)	0.503

Abbreviations: CI = confidence interval, HR = Hazard Ratio.

## 4 Discussion

Adjuvant chemotherapy is considered for patients with stage II SBA with any high‐risk tumor feature or inadequate lymphadenectomy. It is not well understood if an inadequate lymphadenectomy holds prognostic value in the presence or absence of additional high‐risk factors. This study found that among patients with an inadequate lymphadenectomy, those with T4 tumors, poor grade tumor, or positive resection margins were more likely to receive AC. When examining 5‐year overall survival, adjuvant chemotherapy was associated with improved survival among patients with an inadequate lymphadenectomy and any additional high‐risk feature, but not in those without any additional high‐risk features.

Patients with high‐risk features were more likely to receive AC for stage II SBA with inadequate lymphadenectomy, whereas older patients were less likely to receive AC. These high‐risk features included positive resection margin, T4, and poorly differentiated tumors. These results are concordant with retrospective analyses indicating that patients who received chemotherapy for stage II disease were younger and had T4 tumors or inadequate lymphadenectomy [22]. However, AC was administered to just 35.9% of patients with stage II SBA and inadequate lymphadenectomy with at least one additional high‐risk feature, which shows significant treatment heterogeneity. Some patients may have refused chemotherapy, have access or affordability barriers, or been too ill to undergo adjuvant treatment. NCCN guidelines list both observation and adjuvant chemotherapy as management options for patients with stage II SBA with inadequate lymphadenectomy, and thus patients who underwent observation only received guideline‐concordant treatment [14].

Among patients with stage II SBA with a high‐risk feature in addition to inadequate lymphadenectomy, this study found improved survival after receipt of AC in adjusted modeling. The high‐risk features of T4 tumor, lymphovascular invasion, positive margin or poor differentiation suggest more aggressive tumor biology and elevated risk of missed nodal disease. These results are concordant with a previous propensity‐matched analysis of the NCDB found data which supported use of AC in patients with SBA and nodal disease and a potential benefit for those with T4 tumors or positive margins [13]. Likewise, a multicenter retrospective analysis found a survival benefit for AC in patients with stage III disease and for those with high‐risk stage II disease (defined as T4 and/or fewer than 8 lymph nodes examined), suggesting inadequate lymphadenectomy may corelate with disease that would benefit from AC. The present study uses current guideline definitions of inadequate lymphadenectomy and criteria of high‐risk tumor characteristics. The high‐risk SBA tumor characteristics of perineural and lymphovascular invasion or poorly differentiated tumors are based on extrapolation from colorectal studies. When considering the high‐risk features in aggregate, these results suggest that patients with an inadequate lymphadenectomy and any high‐risk feature may have improved outcomes with AC. Notably, fewer than half of such patients received AC in this study's retrospective cohort. For patients with inadequate lymphadenectomy and no additional high‐risk feature, the data indicate numerically higher survival with adjuvant chemotherapy, but this outcome did not reach statistical significance in adjusted modeling. Clinicians may consider individual patient clinical and pathologic factors when recommending adjuvant treatment for a patient with stage II SBA and inadequate lymphadenectomy.

This study has several limitations which affect the ability to address the aims of this study. First, there are limitations associated with utilizing a retrospective database. The NCDB has known selection bias of Commission on Cancer‐associated facilities and underrepresents certain racial and ethnic groups [23]. Second, certain treatment details are not present in the NCDB such as specific regimen and duration of systemic chemotherapy, which may underestimate the effect of adjuvant therapy as patients who complete all cycles of chemotherapy are grouped with those who do not. Third, retrospective studies of postoperative survival are prone to immortal time bias when stratified by an adjuvant treatment, as those who receive the adjuvant treatment must survive to that point to receive it. To adjust for immortal time bias, a landmark analysis was conducted with time zero being 180 days after surgery which showed similar results and association between AC and survival. However, there is likely selection bias in which patients receive adjuvant chemotherapy, such as those who are healthier, which is not fully captured by variables available in the NCDB and may overestimate the beneficial effects of chemotherapy in this retrospective analysis. Fourth, data on disease‐specific recurrence is not available, and limits the ability to assess recurrence‐free survival after curative‐intent surgery for stage II SBA.

## 5 Conclusion

Among patients with upfront resection for stage II small bowel adenocarcinoma and an inadequate lymphadenectomy, 29.8% received adjuvant chemotherapy and 57.4% had an additional high‐risk feature such as T4 tumor, poorly differentiated tumor, lymphovascular invasion, or positive resection margin. Patients were more likely to receive adjuvant chemotherapy if they had a T4 tumor, poor grade tumor, or positive resection margins. Receipt of AC was associated with improved 5‐year postoperative survival in patients with inadequate lymphadenectomy and any additional high‐risk feature but not in patients with no additional high‐risk feature. Multiple patient and pathologic variables should be considered in decisions regarding AC in this patient population.

## Conflicts of Interest

The authors declare no conflicts of interest.

## Synopsis

Among patients with upfront resection for stage II small bowel adenocarcinoma, 35% had an inadequate lymphadenectomy. Receipt of adjuvant chemotherapy was associated with improved 5‐year postoperative survival in patients with inadequate lymphadenectomy and any additional high‐risk feature (such as T4 tumor, poorly differentiated tumor, lymphovascular invasion, or positive resection margin) but not in patients with no additional high‐risk feature. Decisions regarding chemotherapy should consider multiple patient and pathologic variables.

## Supporting information


**Supplemental Table 1:** Cox proportional hazard ratios for mortality via landmark analysis beginning 180 days after surgery among patients with pathologic stage II small bowel adenocarcinoma and inadequate lymphadenectomy.

## Data Availability

The data used in the study are derived from a de‐identified NCDB file. The American College of Surgeons and the Commission on Cancer have not verified and are not responsible for the analytic or statistical methodology employed, or the conclusions drawn from these data by the investigator. Dr. Baril is supported by the National Cancer Institute of the National Institutes of Health under Award Number T32CA282070. The content is solely the responsibility of the authors and does not necessarily represent the official views of the National Institutes of Health.
