# Supplementary material for: Treatment and Outcomes for Patients With Inadequate Lymphadenectomy After Resection of Stage II Small Bowel Adenocarcinoma
Source: J Surg Oncol. 2025 Dec 9;133(2):186–95. doi: 10.1002/jso.70151 (PMC12863234; doi:10.1002/jso.70151)
Supplement: Supplementary file 1 — Supplemental Table 1: Cox proportional hazard ratios for mortality via landmark analysis beginning 180 days after surgery among patients with pathologic stage II small bowel adenocarcinoma and inadequate lymphadenectomy. [file JSO-133-186-s001.docx]

Supplemental Tables & Figures:

Supplemental Table 1: Cox proportional hazard ratios for mortality via landmark analysis beginning 180 days after surgery among patients with pathologic stage II small bowel adenocarcinoma and inadequate lymphadenectomy. Patients are stratified by presence or absence of at least one additional high-risk feature.

|  | With Additional  High-Risk Feature(s)  N=557  HR (95% CI) | P value | Without Additional  High-Risk Feature  N=476  HR (95% CI) | P value |
| --- | --- | --- | --- | --- |
| *Patient and Tumor Characteristics* |  |  |  |  |
| Post-operative Chemotherapy  No  Yes | 1  0.72 (0.54-0.95) | Ref  0.018 | 1  0.87 (0.57-1.31) | Ref  0.501 |
| Sex  Female  Male | 0.72 (0.55-0.93)  1 | 0.011  Ref | 1.32 (0.96-1.82)  1 | 0.088  Ref |
| Age (yrs)  <50  50-59  60-69  70-79  80+ | 1.00 (0.58-1.70)  1  1.34 (0.89-2.01)  1.17 (0.72-1.89)  1.73 (1.03-2.92) | 0.986  Ref  0.155  0.535  0.038 | 0.62 (0.30-1.31)  1  0.86 (0.50-1.47)  0.99 (0.54-1.82)  2.19 (1.19-4.01) | 0.211  Ref  0.577  0.983  0.011 |
| Race and Ethnicity  Non-Hispanic White  Non-Hispanic Black  Hispanic  Asian  Other or Unknown | 1  1.32 (0.92-1.90)  1.31 (0.69-2.47)  0.49 (0.11-2.12)  1.92 (1.21-3.05) | Ref  0.134  0.410  0.336  0.006 | 1  1.13 (0.74-1.72)  1.57 (0.69-3.58)  1.52 (0.35-6.72)  1.38 (0.69-2.75) | Ref  0.578  0.283  0.578  0.360 |
| Median Household Income  <$46,277  $46,277 - $57,856  $57,857 - $74,062  $74,063+ | 1  0.72 (0.46-1.14)  0.83 (0.54-1.29)  0.60 (0.37-0.97) | Ref  0.162  0.441  0.038 | 1  1.05 (0.60-1.83)  1.05 (0.59-1.86)  1.45 (0.78-2.72) | Ref  0.874  0.868  0.242 |
| % Local No High School Diploma  <5%  5.0-9.0%  9.1-15.2%  15.3% and up | 1  0.66 (0.45-0.96)  0.54 (0.34-0.85)  0.58 (0.35-0.96) | Ref  0.162  0.411  0.035 | 1  0.93 (0.56-1.56)  1.19 (0.67-2.11)  0.80 (0.40-1.60) | Ref  0.790  0.544  0.530 |
| Insurance Status  Private Insurance  Medicare  Uninsured/Medicaid  Other/Unknown | 1  0.98 (0.68-1.42)  1.35 (0.82-2.23)  0.96 (0.44-2.12) | Ref  0.906  0.232  0.936 | 1  1.44 (0.91-2.29)  2.14 (1.15-3.99)  4.01 (1.64-9.75) | Ref  0.123  0.016  0.002 |
| Charlson-Deyo Score  0  1  2+ | 1  1.44 (1.06-1.96)  2.23 (1.48-3.38) | Ref  0.020  <0.001 | 1  1.20 (0.81-1.76)  1.43 (0.82-2.49) | Ref  0.364  0.207 |
| Year of Diagnosis  2004-2008  2009-2013  2014-2018  ≥2018 | 1.40 (0.73-2.67)  1.08 (0.65-1.80)  1.08 (0.66-1.79)  1 | 0.310  0.756  0.757  Ref | 1.27 (0.60-2.69)  1.03 (0.49-2.18)  0.99 (0.46-2.13)  1 | 0.523  0.929  0.987  Ref |
| Tumor Location  Duodenum  Jejunum  Ileum  Other, Small Intestine | 1  0.75 (0.47-1.17)  0.94 (0.58-1.54)  1.18 (0.76-1.83) | Ref  0.201  0.814  0.448 | 1  0.66 (0.40-1.10)  0.72 (0.40-1.30)  1.12 (0.69-1.81) | Ref  0.111  0.273  0.652 |
| *Hospital Characteristics* |  |  |  |  |
| Hospital Type  Academic  Non-Academic | 1  1.12 (0.78-1.60) | Ref  0.534 | 1  1.47 (0.94-2.29) | Ref  0.090 |
| Hospital Annual Volume Quartile  Q1 (<4)  Q2 (4-8)  Q3 (9-15)  Q4 (≥16) | 0.81 (0.52-1.27)  0.90 (0.0.60-1.37)  0.84 (0.56-1.28)  1 | 0.365  0.627  0.422  Ref | 1.01 (0.35-1.91)  1.43 (0.78-2.60)  0.85 (0.46-1.58)  1 | 0.987  0.243  0.605  Ref |
| Hospital Region  New England  Middle Atlantic  South Atlantic  East North Central  East South Central  West North Central  West South Central  Mountain  Pacific | 1  1.02 (0.52-2.01)  1.08 (0.54-2.13)  0.74 (0.40-1.48)  0.80 (0.37-1.74)  1.12 (0.55-2.28)  0.91 (0.44-1.91)  1.20 (0.49-2.95)  0.81 (0.39-1.68) | Ref  0.959  0.824  0.394  0.570  0.757  0.810  0.685  0.575 | 1  1.81 (0.68-4.83)  1.36 (0.52-3.59)  1.95 (0.73-5.19)  1.28 (0.43-3.81)  2.20 (0.77-6.34)  1.52 (0.53-4.36)  1.40 (0.38-5.16)  1.32 (0.46-3.76) | Ref  0.238  0.534  0.180  0.660  0.143  0.436  0.615  0.601 |
